# Enhancing Attention and Interest in Plants to Mitigate Plant Awareness Disparity

**DOI:** 10.3390/plants12112201

**Published:** 2023-06-02

**Authors:** Pavol Prokop, Jana Fančovičová

**Affiliations:** 1 Department of Environmental Ecology and Landscape Management, Faculty of Natural Sciences, Comenius University, 842 15 Bratislava, Slovakia; 2 Institute of Zoology, Slovak Academy of Sciences, 845 06 Bratislava, Slovakia; 3 Faculty of Education, Trnava University, Priemyselná 4, 918 43 Trnava, Slovakia; jana.fancovicova@truni.sk

**Keywords:** attitudes toward plants, flower attractiveness, plant blindness, pollinators and science education pollinators and science education, willingness to protect

## Abstract

Plant awareness disparity (PAD, formerly plant blindness) is the human inability to notice plants in everyday life. It is suggested that the main underlying factors of PAD are: 1. the inability to recognize individual plants and 2. stronger preferences for animals, which prevents building positive attitudes toward them. The presentation of individual plants should trigger more positive responses toward them than the presentation of groups of plants. Strong preferences for animals predict that the presence of an animal on a plant might enhance positive perceptions of the plant by people. We experimentally investigated the perceived attractiveness and willingness to protect (WTP) plants presented individually and in groups and with or without various pollinators in a sample of Slovak people (*N* = 238). In contrast to the first prediction, only one of four plants (dog rose, but not saffron, spruce, or beech tree) received higher attractiveness scores when presented individually than in a group. None of these species received higher WTP scores when presented individually, rather than in a group. The effect of the presence of pollinators on flower attractiveness and WTP was distinguished between vertebrates and invertebrates; only flowers with birds and bats increased their attractiveness scores, while flowers with invertebrates, including a butterfly, honeybee, beetle, and the syrphid fly, received similar or lower scores than the same plant species without pollinators. WTP plants significantly increased only when the scarlet honeycreeper and the cave nectar bat were present on flowers as pollinators. People showed significantly stronger preferences for items that associate 1. plants with pollinators and 2. plants with animals that distribute animal seed than for items focused solely on plants. Connecting animals and plants should help reduce PAD. This aim cannot be achieved, however, by presenting individual plants and/or plants with randomly chosen pollinators.

## 1. Introduction

All species in the order of primates, including humans, depend predominantly on plant food [1]. After the transition of hunter-gatherers to farmers about 10,000 years ago [2], our dependency on plants, which were cultivated and selected for their nutritional qualities, increased due to the rapid population growth of the human population [3]. Knowledge regarding plant cultivation and determination were crucial for survival and transmitted between generations [4,5]. Paradoxically, rapid technological change and drastic reduction in the rural population [6,7] prohibit daily activities of crop cultivation and reduce regular contact with nature, leading to progressive loss of human–nature interactions [8,9,10].

Plant awareness disparity (PAD [11]), formerly known as plant blindness [12,13], is defined as the tendency not to notice plants in our environment, leading to the devaluation of plants, concerning their crucial role in ecosystem functioning, environmental sustainability, public health, and ultimately the survival of humans on Earth [14,15,16]. PAD is determined by cultural and evolutionary factors [17]. Humans group plants into uniform green masses, which prevents them from appreciating the unique biological features of plants and, instead, strongly prefer animals over plants [18,19,20,21,22,23,24]. University students, for instance, showed better recall of animal names than plant names, even though they were equally nameable [20]. Similar results were obtained with research on primary children [25]. It was shown that the use of individual images of animals and plants, presented in rapid succession, resulted in better recall tasks of animals than plants [26,27,28]. This suggests that the human visual system is tuned to respond predominantly to things that move and/or look similar to us to avoid potential predators or localize food sources or conspecific mating partners or competitors [29]. Indeed, recall success in plant names significantly increases when a plant’s survival value is considered [30,31]. 

PAD has non-negligible consequences on policy on illegal wildlife trade and a more widespread impact on conservation science [22,24,32]. Scientists publish fewer papers focused on plants in conservation journals compared to papers about animals [33], and research on plants is biased toward blue-coloured plants, irrespective of their conservation status [34]. Lower attention to plants is consequently translated into lower funding in conservation than animals [17,35,36], despite plant extinction reaching its maximal peak in written human history [37]. Alarmingly, less than 8% of all known plant species are assessed by the IUCN red list, compared with 68% of all known vertebrates [32]. 

PAD has four domains: attention, attitude, knowledge, and relative interest. The first domain refers to the attention people devote to plants. Attitude concerns feeling about plants, and knowledge refers to understanding their role in nature. Relative interest includes how people find plants interesting compared with animals and whether they are willing to protect them [11,38]. Attention, attitude, and relative interest are predominantly affective domains, while knowledge is purely cognitive. In this paper, we implicitly and explicitly investigated the three affective domains because of their crucial role in nature conservation [39,40,41,42,43,44,45]. It is suggested that organisms which capture human attention (e.g., by colour) also enhance their willingness to protect them [41,46,47]. More positive attitudes and interest in living organisms support human willingness to pay for their conservation [39,48]. 

We manipulated the number of plants and the presence of animals next to the plants in the pictures. We examined their aesthetic value (attitude domain) and willingness to protect them (relative interest domain). The inability of individual recognition of plants is one of the factors underlying plant blindness [11,12,49]. We hypothesize that plants presented individually are more aesthetically appealing to people who are consequently more prone to protect them. Given that presenting animals next to plants increases the recall scores of plants [28], we hypothesize that plant flowers with their pollinators trigger the aesthetic value of plants and contribute to the willingness to protect these plants. In line with this hypothesis, we further predicted that items showing connections between animals and plants are perceived to be more attractive to people than items solely about plants. Finally, females are more attentive to plants than males [20,50,51,52,53]; thus, we predict that attractiveness and willingness to protect plants are higher in females than in males. 

## 2. Results

### 2.1. Are Individual Plants Perceived More Positively Than Plants in Groups?

The perception of the attractiveness of individual plants and the same plants in groups was not uniform. Only the dog rose presented individually was perceived as more attractive than in a group (Table 1). Both saffron and spruce were perceived as more attractive in groups. The beech tree presented individually, and, in a group, received a similar score. In terms of willingness to protect plants, however, spruce presented individually received significantly higher WTP scores than in the group (Table 1). Other differences were not significant. These results do not support the hypothesis that plants presented individually are more aesthetically appealing and that people are more willing to protect them. The mean attractiveness scores of the plants presented individually were significantly correlated with WTP scores of plants presented individually (Spearman *r* = 0.44, *p* < 0.001). A similar correlation was found between attractiveness and WTP plants presented in groups (Spearman *r* = 0.45, *p* < 0.001). 

### 2.2. Are Plants Presented on Their Own Perceived Differently Than Those with Pollinators?

**Figure 1:**
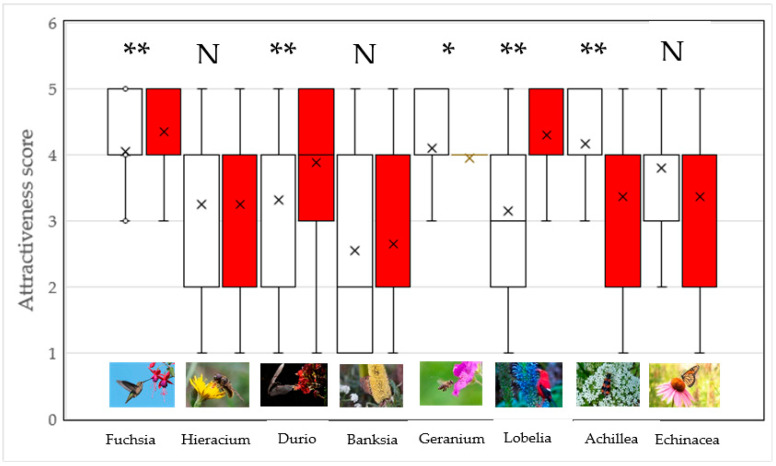
The perception of the attractiveness of plant flowers presented on their own (open boxes) and with pollinators (red boxes). The asterisks denote significant differences (* *p* < 0.05, *** *p* < 0.001) based on the Wilcoxon-matched pairs test. N = not significantly different. Box plots represent medians, means (x), 25th and 75th percentiles, as well as minimum and maximum values.

Perception of the attractiveness of plants with and without pollinators was not uniform (Figure 1). The perceived attractiveness of the flowers increased through the presence of pollinators in *Fuchsia*, *Durio*, and *Lobelia* (Figure 1). The pollinators of these plants were the hummingbird, cave nectar bat, and scarlet honeycreeper, respectively. The presence of the honey bee and soldier beetle significantly decreased the attractiveness of *Geranium* and *Achillea*, respectively. Hoverflies, Monarch butterflies and honey possums had no significant influence on the attractiveness of *Hieracium*, *Echinacea*, and *Banksia* flowers (Figure 1). 

**Figure 2:**
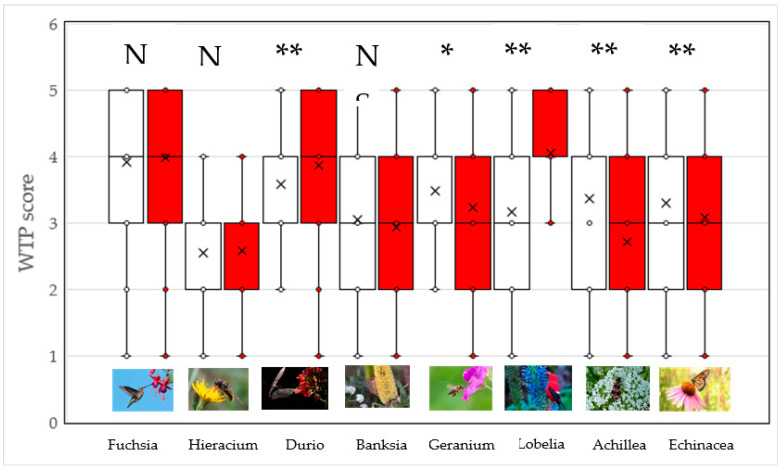
WTP of plants presented on their own (open boxes) and with pollinators (red boxes). The asterisks denote significant differences (* *p* < 0.05, ** *p* < 0.01) based on the Wilcoxon-matched pairs test. N = not significantly different. Box plots represent medians, means (x), 25th and 75th percentiles, as well as minimum and maximum values.

Regarding willingness to protect plants, *Durio* and *Lobelia* increased their scores due to the presence of their pollinators (cave nectar bat and scarlet honeycreeper, respectively). In contrast, the presence of the honeybee, the soldier beetle, and the Monarch butterfly on *Geranium*, *Achillea*, and *Echinacea* significantly decreased WTP scores (Figure 2), respectively. Other differences were not significant. These results provide only partial support for the hypothesis that plant flowers presented with their pollinators trigger the aesthetic value of plants and contribute to the willingness to protect these plants. In fact, the plausibility of this hypothesis is restricted to specific vertebrate pollinators, particularly to the cave nectar bat and scarlet honeycreeper. The mean scores between perceived attractiveness and WTP of plants presented on their own significantly correlated (Spearman *r* = 0.40, *p* < 0.001). Similarly, the mean scores of perceived attractiveness and WTP of plants presented with pollinators significantly correlated (Spearman *r* = 0.52, *p* < 0.001).

### 2.3. Are There Differences in the Perception of Plants between Males and Females?

Regarding the four plant species presented individually or in groups (the list of species is shown in Table 1), both saffron and dog rose received significantly higher attractiveness scores from females than from males (M-W U-tests with Bonferroni corrected *p*-values, all *p* < 0.001, data not shown). When the saffron was presented individually, females showed higher WTP scores than males. When dog roses were presented in the group, females again showed higher WTP scores than males (M-W U-tests, *p* < 0.001 and 0.012, respectively). In contrast, the saffron presented in the group received similar WTP scores between the sexes. The individual dog rose also received similar WTP scores with respect to gender (M-W U-tests, *p* = 0.04 and 0.33, respectively). There were no gender differences in perceived attractiveness and WTP for the spruce and beech tree. 

### 2.4. The Influence of Pollinators on Differences between Males and Females 

In all but one case, females showed higher attractiveness scores than males in *Hieracium, Geranium, Achillea*, and *Echinacea* presented both solely and with pollinators (M-W U-tests with Bonferroni corrected *p*-values, all *p* < 0.025 and less). Only *Lobelia* presented with the scarlet honeycreeper was significantly more preferred by males (*p* = 0.02). There were no significant gender differences in the WTP at the species level in these eight plant species (a list of all species can be found in Figure 1 and Figure 2).

### 2.5. Participants’ Preferences for Animal-Plant Interaction Topics

There were significant preferences for animal–plant interaction topics in four out of five items described in detail below. Participants showed the strongest preference for honey production from nectar (74%), seed dispersal mediated by bats (70%), plant pollination by birds (70%), and the production of special substances used by orchids for the deceptive attraction of pollinators (57%) (binomial tests, *p* < 0.001, 0.001, 0.001 and 0.05, respectively). Only the production of colourful flowers aimed at pollinator attraction showed a preference not significantly different by chance (55%, binomial test, *p* = 0.17). 

## 3. Discussion

This study experimentally examined the affective domains of PAD [11,38] to show how we can improve people’s interest in plants. We implicitly assumed that the presence of pollinators next to plants increases the participant’s attention, which consequently positively influences his/her attitudes to and interest in the plant through increased perceived attractiveness and WTP. We similarly thought that, when a plant is presented individually rather than in a group, participants will consider the plant more attractive, and they will be more prone to protect it. Unfortunately, both hypotheses remained largely unsupported because no consistent changes in participant ratings of plants were observed. The only exceptions were vertebrate pollinators, with the majority of species (except for the the honey possum) contributing to plant attractiveness. Since the same plant species were presented with or without pollinators (or individually vs. in groups), other plant attributes, such as colour or morphology, do not confound these results. 

Our first hypothesis dealt with the superficial visual perception of plants as a green mass instead of a cluster of several individuals e.g., [49], which prevents emotional bonding between humans and plants. This hypothesis was not supported because only one of the four species (dog rose) received a significantly higher attractiveness score when presented individually, rather than in a group. In contrast, individual saffron received a very high attractiveness score, which grew when presented in the group. Furthermore, the presence of flowers increases their attractiveness to humans [45,54]. Therefore, the increased abundance of attractive flowers would further contribute to their overall attractiveness. The saffron in our study had purple flowers, and purple is a mix of red and blue colours. Both these colours are attractive to humans [30,36,55]. In contrast, the dog rose in our study was of less saturated pink/white colours, in all probability less attractive to the participants. The high abundance of less attractive dog rose flowers reduced their self-perceived attractiveness. 

The spruce was perceived to be more attractive in a group than individually, suggesting a positive attitude of the participants toward the woods, rather than individual trees. It remains to be seen, however, why the same pattern has not been observed for the beech tree and why the (more attractive) spruce trees presented in the group received lower mean WTP scores than the individual. Overall, individual plants received higher mean WTP scores than those presented in groups, which supports the idea that humans prefer to favour rare species [56,57,58]. This effect did not receive, however, statistical significance in most species (3/4). 

Plants presented next to animals received better recall [28], and it was expected that the presence of animals in pictures would contribute to plant attractiveness and WTP. We found that the animal effect was equivocal because only three out of eight pollinators (namely, the scarlet honeycreeper, cave nectar bat, and Lucifer hummingbird) contributed to plant attractiveness, and only two out of eight significantly increased plants’ WTP scores. Zani and Low [28] experimentally manipulated the presence of large mammals (e.g., horses, cows) next to plants and did not investigate the affective domains of plant perception by people. Large, charismatic mammals attract people [33,59,60,61] uncomparably more than insects [62,63,64,65], the most common pollinators of plants. Unlike other vertebrate pollinators in this study, the honey possum did not influence the attractiveness and WTP plants. The honey possum resembles small rodents, which are not typically considered attractive by people [62,66].

Scarlet honeycreeper and the Lucifer hummingbird are species with apparent colouration that attract people [67,68]. However, the positive influence of the cave nectar bat remains a mystery, since bats do not have a good reputation among people, e.g., [58,62,63]. Perhaps people have limited knowledge about the role of bats as pollinators [69], and this “surprise effect” resulted in a more positive evaluation (attractiveness and WTP) of Durian Nyekak. Another surprise is that the presence of the honey bee and the soldier beetle significantly decreased flower attractiveness and WTP. The presence of the Monarch butterfly decreased the WTP, but it showed no influence on the attractiveness of the plant. Butterflies are attracted to people due to their beautiful colours [62,63,65,70], bees are positively associated with ecosystem services, such as honey production and pollination [70], and the soldier beetle has a conspicuous, aposematic colour, which enhances WTP [41]. We suggest that these unexpected results could be partly explained by the fear of being stung by a honeybee [71]. Perhaps bumblebees have a better reputation than honeybees [62,65,72,73], and they can be used in future studies instead of honeybees. More familiar species could replace butterflies and beetles because people are more willing to protect native instead of non-native species [74]. 

If invertebrate pollinators showed a largely negative or neutral influence on plant attractiveness and WTP plants, it is a question for future research whether attractive plants influence human perception of pollinators. Perhaps flowers can have a positive impact on attitudes toward insect pollinators more than when pollinators are presented alone, cf. [75]. 

Gender differences were, in part, in line with our hypotheses; females preferred plants with flowers more than those without flowers [45]. Prokop and Fančovičová [55] similarly found more significant aesthetic preferences for fruit by females. Females were less prone, however, to protect plants than males, which contradicts our hypothesis. Perhaps females would be more experienced with the presented plants and could know that many of them (e.g., dog rose, spruce tree) do not need urgent protection. 

Significant preferences of participants toward animal–plant interaction topics would seem to be promising opportunities for mitigating PAD and building people’s positive attitudes toward nature in general. Although visual manipulation of the presence of pollinators did not bring the expected result, animal–plant interactions are of interest to people and need to be guided by science teachers and media. The problem is that only a few studies have explored people’s perception of multiple insect and non-insect pollinators, such as birds or bats [69]. It is, therefore, difficult to predict people’s responses. Moreover, there are no correlations between interest in plants and animals [23,50], meaning that animals and plants are distinct organisms for laypeople. Science education activities and field trips need to support people’s natural curiosity toward animal–plant interactions. 

Our research was based on affective domains underlying people’s emotional connection with plants (attention, attitude, and relative interest). These domains correspond with the development of values, necessarily supporting an individual’s concern for the environment and building human–nature relationships. Creating an individual’s connectedness to nature is one of the goals of environmental education [76,77]. Our results showed that connections between vertebrate pollinators (except for honey possum) and plants enhance plant attractiveness and individual WTP plants in the cave nectar bat and honeycreeper. Using these unusual examples of pollinators might enhance learner’s emotional connections with both animal and plant needs and, consequently, concerns for the rapidly changing environment and ecosystem degradation. Science curricula and textbook/workbook development should be informed about the positive influences of specific pollinators on plant attractiveness/WTP. We are not suggesting, however, that native species, particularly arthropod pollinators, should be overlooked; instead, we call for a simultaneous interplay between building positive attitudes toward invertebrates and plants and avoiding the exclusive use of charismatic animals [78,79]. 

## 4. Materials and Methods

### 4.1. Participants

The participants were *N* = 238 Slovaks (178 females) aged between 10 and 54 years of age (mean age = 18, SD = 7.28). The study was conducted online. Participants were recruited for the study online via the university website. Additionally, ten science teachers were asked to kindly recruit volunteers from their schools to collect data from school-aged participants. The selection of participants was not limited by gender or age because we intended to collect data from diverse samples. School-aged participants received a link to a research questionnaire (prepared in Google Forms) from their teachers. All the participants were blind to our research hypotheses, and the research was anonymous. Ethical approval was obtained from the Institutional Board of Trnava University in accordance with the principles of the Declaration of Helsinki. All participants provided informed consent before completing the survey.

### 4.2. Selection of Pictures

We used colour pictures of 12 plant species freely downloaded from Google. Latin or English names were used when searching for each plant species. When searching for pictures of pollinators on plants, we used the names of the pollinators (e.g., ‘cave nectar bat pollinating’) instead of the species names. 

### 4.3. Individual Plants vs. Plants in Groups

Four species were used to manipulate the number of plants (Table 1). Specifically, plants were presented in four pictures in isolation (i.e., one specimen), and the other four pictures showed the same plant species in a group (e.g., one spruce vs. spruce wood). Herbs (saffron) and bushes (dog rose) were shown with flowers because flowers could help participants identify individuals from groups. Trees (beech tree and spruce) were shown without flowers because humans use their trunks, rather than inconspicuous flowers, for individual identification. 

### 4.4. Manipulation of the Presence of Pollinators

Eight plant species with their flowers (for a list of species, see Figs. 1, 2) were used to manipulate the presence of pollinators on their flowers. We aimed to examine various species of pollinators; thus, each plant species was presented with different pollinator species. For example, fuchsia (*Fuchsia hybrida* ‘Multa’) was presented with (and without) the Lucifer hummingbird *(Calothorax lucifer*, Swainson, 1827), and the wall hawkweed (*Hieracium murorum*, Linnaeus, 1753) was presented with (and without) hoverfly *Cheilosia canicularis* (Panzer, 1801). Durian Nyekak (*Durio kutejensis*, Hasskarr, 1858) was presented with (and without) the cave nectar bat (Eonycteris spelaea, Dobson, 1871), and the candlestick banksia (*Banksia attenuata*, Brown, 1810) was presented with (and without) the honey possum (*Tarsipes rostratus*, Gervais and Verreaux, 1842). The wood cranesbill (*Geranium sylvaticum*, Linnaeus, 1753) was presented with (and without) the honeybee (*Apis mellifera*, Linnaeus, 1758), and Gray’s Lobelia (*Lobelia grayana*, Wimmer, 1845) was presented with (and without) the scarlet honeycreeper (*Vestiaria coccinea*, Forster, 1780). Finally, the common yarrow (*Achillea millefolium*, Linnaeus, 1753) was presented with (and without) the soldier beetle (*Trichodes alvearius*, Fabricius, 1792), and the coneflower echinacea (*Echinacea purpurea* [L.], Moench, 1794) was presented with (and without) the monarch butterfly (Danaus plexippus, Linnaeus, 1758). 

### 4.5. Measuring Preferences for Animal–Plant Interactions

Participants’ preferences for animal–plant interactions were examined with five pairs of short items self-constructed by the authors. Participants were asked to choose only one of each pair of items: 1. “Pollination of plants” or “Pollination of plants by birds”; 2. “Transfer of plant seeds” or “Transfer of plant seeds by bats”; “The formation of colourful plant flowers” or “The formation of colourful plant flowers that attract pollinators”; “Production of nectar in flowers” or “Production of nectar in flowers from which bees make honey”; and “Production of special substances in orchids” or “ Production of special substances in orchids with which they falsely attract and deceive pollinators”. We intentionally avoid the term “seed dispersal” and “pheromones” (items no. 2 and 5, respectively) because they might not be clearly understood by laypeople. 

### 4.6. Measures

At the beginning of the questionnaire, participants were asked about their age and sex. Then, each plant in the picture was rated on self-perceived attractiveness (dependent variable) (“How attractive would you consider this plant?” 1 = not at all, 5 = extremely attractive) on a 5-point scale. Another dependent variable, the willingness to protect plants (WTP), was defined as support for protection of a specific plant species, also on a 5-point scale (“Do you think that this plant should be protected by laws?” 1 = not necessary to protect, 5 = extremely important its protection) following similar research [41,47]. The pictures were presented in random order. 

### 4.7. Statistical Analyses

This research used within-subject design, which involves testing the same group of participants under different conditions (e.g., the same plant species with and without a pollinator). A strong advantage of within-subject designs is that they control for individual differences between participants, which can increase the sensitivity of the study. In addition, within-subject design often requires fewer participants than between-subject designs to achieve similar levels of statistical power [80]. Data were not normally distributed. We, therefore, used non-parametric tests. The Mann-Whitney U-test was used to examine differences in perceived attractiveness and WTP scores for independent groups (e.g., gender), and the Wilcoxon-matched pairs test was used to compare scores in the same participants (e.g., a flower presented with and without a pollinator). *p*-values for gender differences were Bonferroni corrected (α = 0.05/2) to avoid Type I error. The correlations between the variables were performed with Spearman rank correlation coefficients. Correlations between all variables examined and age were negligible; we, therefore, do not refer to age-related trends. Participants’ preferences for items focused on animal–plant interactions were calculated with the binomial test. All statistical tests were performed with IBM SPSS Statistics for Windows, ver. 26.0 [81]. 

## 5. Conclusions

PAD is a pervasive phenomenon with broad negative consequences. Our research failed to support the idea that presenting individual plants (instead of plants in groups), to prevent viewing plants as a green mass, promotes affective commitment to plants, except for plants people considered beautiful. People are naturally curious to know more about animal–plant relationships than plants alone. Although this is further evidence of the existence of PAD, this finding can be used to mitigate the low interest of people in plants. Efforts should be directed to include non-insect pollinators in science education lessons because they can help us increase natural curiosity in both animals and, consequently, in plants. Science teachers should guide these activities because visual material containing pollinators (mainly insects) is not helpful enough to increase people’s affective domains of PAD.

## Figures and Tables

**Table 1:** Median scores (±95% CI) for perceived attractiveness and willingness to protect (WTP) plants presented individually and in groups.

		Saffron (*Crocus* sp.)	Dog Rose (*Rosa canina* Linnaeus, 1753)	Spruce (*Picea abies* Linnaeus, 1753)	Beech Tree (*Fagus sylvatica* Linnaeus, 1753)
Attractiveness	Individually	4 (4.13, 4.33)	4 (3.32, 3.61)	4 (3.38, 3.65)	4 (4.22, 4.42)
	In group	5 (4.31, 4.5)	4 (3.04, 3.35)	4 (3.72, 3.98)	4 (4.15, 4.37)
	Wilcoxon Z	3.25	4.32	4.47	0.90
	*p*	0.001	<0.001	<0.001	0.37
WTP	Individually	4 (3.92, 4.18)	3 (2.68, 2.99)	3 (2.66, 2.99)	4 (3.49, 3.81)
	In group	4 (3.8, 4.07)	3 (2.61, 2.92)	3 (3.12, 3.46)	4 (3.34, 3.68)
	Wilcoxon Z	1.44	1.12	5.5	1.24
	*p*	0.15	0.26	<0.001	0.21

## Data Availability

Data and visual materials are available from the corresponding author upon request.
